# When unconscious rewards boost cognitive task performance inefficiently: the role of consciousness in integrating value and attainability information

**DOI:** 10.3389/fnhum.2012.00219

**Published:** 2012-07-25

**Authors:** Claire M. Zedelius, Harm Veling, Henk Aarts

**Affiliations:** Department of Psychology, Utrecht UniversityUtrecht, Netherlands

**Keywords:** rewards, conscious and unconscious processing, attainability, motivation, cognitive control, performance

## Abstract

Research has shown that high vs. low value rewards improve cognitive task performance independent of whether they are perceived consciously or unconsciously. However, efficient performance in response to high value rewards also depends on whether or not rewards are attainable. This raises the question of whether unconscious reward processing enables people to take into account such attainability information. Building on a theoretical framework according to which conscious reward processing is required to enable higher level cognitive processing, the present research tested the hypothesis that conscious but not unconscious reward processing enables integration of reward value with attainability information. In two behavioral experiments, participants were exposed to mask high and low value coins serving as rewards on a working memory (WM) task. The likelihood for conscious processing was manipulated by presenting the coins relatively briefly (17 ms) or long and clearly visible (300 ms). Crucially, rewards were expected to be attainable or unattainable. Requirements to integrate reward value with attainability information varied across experiments. Results showed that when integration of value and attainability was required (Experiment 1), long reward presentation led to efficient performance, i.e., selectively improved performance for high value attainable rewards. In contrast, in the short presentation condition, performance was increased for high value rewards even when these were unattainable. This difference between the effects of long and short presentation time disappeared when integration of value and attainability information was not required (Experiment 2). Together these findings suggest that unconsciously processed reward information is not integrated with attainability expectancies, causing inefficient effort investment. These findings are discussed in terms of a unique role of consciousness in efficient allocation of effort to cognitive control processes.

Motivation is an essential determinant of cognitive control and performance (Watanabe, 2007). Accordingly, a vast body of research has studied how rewards affect cognition and behavior (Wood et al., 1999). Whereas the neuro-cognitive processes underlying the effects of rewards on human cognition and behavior are not yet entirely understood (Chiew and Braver, 2011), it has become clear that the anticipation of rewards can cause people to increase their effort and performance on various cognitive and behavioral tasks (Brehm and Self, 1989; Camerer and Hogarth, 1999; Bonner and Sprinkle, 2002).

Most research on the effects of rewards on the control of cognition and behavior has focused on consciously communicated rewards. In these studies people are fully aware of the specific reward that can be gained through optimal performance on a task. However, research on unconscious processes in the motivation and control of goal-directed behavior challenges the assumption that conscious awareness of rewards is necessary to boost performance of cognitive control or working memory (WM) processes (Nieuwenhuis et al., 2001; Hassin et al., 2009; Bargh et al., 2010; Custers and Aarts, 2010; van Gaal et al., 2010). For instance, studies have shown that high compared to low rewards boost performance on WM tasks even when they are presented unconsciously (for a review see Bijleveld et al., 2012). This intriguing finding offers a new direction to understanding how rewards affect the control of human cognition and behavior, raising the question of whether conscious reward processing plays a unique role in modulating cognitive performance. In the present study, we aim to explore this issue by investigating how people deal with attainable and unattainable monetary rewards when such rewards are consciously or unconsciously processed.

It has long been recognized that presenting valuable rewards does not necessarily improve task performance (e.g., Hull, 1943; Brehm and Self, 1989). An important factor in determining whether a reward will boost performance is whether the reward is perceived as attainable. Studies addressing the expected value analysis of human decision making have found that when attainability information is provided, people no longer base their decisions to invest effort on the reward value alone, but on the combination of value and attainability (von Neumann and Morgenstern, 1947; Atkinson, 1957, 1964; Vroom, 1964; Brehm and Self, 1989; Camerer and Hogarth, 1999; Bonner and Sprinkle, 2002). Performance increases when a reward is both valuable and attainable, but is reduced whenever a reward is of low value or unattainable. This finding is consistent with the general notion that people are conservative in spending their valuable mental resources (Kool et al., 2010; Gendolla et al., 2012). Thus, from the above studies, it appears that people readily integrate the value of a reward with attainability information in order to avoid wasting effort. However, participants in these studies were always aware of the value of a reward at stake and the potential influence of this reward on their performance. In light of work suggesting that reward pursuit can occur outside of awareness (for a review see Custers and Aarts, 2010), we investigated the question of how cognitive performance is affected by the value of an unconsciously perceived reward in a context where the reward is unattainable.

Recently, researchers have developed an experimental paradigm that allows the examination of this question. In this paradigm, participants are presented with coins of high and low value which can be attained as rewards for successful performance on a task. Importantly, on half of the experimental trials the reward is presented unconsciously (i.e., subliminally), whereas the rewards are consciously visible (i.e., supraliminally presented) on the other trials. This procedure enables the direct comparison of the effects of conscious and unconscious reward processing on task performance. Using this paradigm, studies have shown parallel effects of conscious and unconscious reward presentation. For instance, in the first study employing this paradigm (Pessiglione et al., 2007) participants could gain rewards by squeezing a handgrip. Not surprisingly, high vs. low value rewards resulted in harder squeezing. Remarkably, people still squeezed harder for more valuable rewards when these were presented subliminally. Other studies have found enhanced mental effort and performance through consciously and unconsciously-presented high rewards on executive control and WM tasks, such as active maintenance and updating of ordered information (Bijleveld et al., 2009; Capa et al., 2011; Zedelius et al., 2011b; Bustin et al., 2012). However, there have also been studies showing that conscious and unconscious rewards in some task contexts can lead to different effects (e.g., Bijleveld et al., 2010, 2011; Zedelius et al., 2011b).

Recently, a theoretical framework has recently been proposed to account for both identical and divergent effects of conscious and unconscious rewards on performance. This framework distinguishes initial (or unconscious) reward processing from full (or conscious) reward processing (Bijleveld et al., 2012). According to this framework, people initially process rewards in rudimentary brain structures that respond to the value of rewards and boost task performance directly by causing increased recruitment of effort. This process is thought to operate without requiring conscious awareness, which explains why unconsciously perceived rewards can enhance performance. After initial reward processing, when rewards are consciously perceived (e.g., by prolonging presentation time from subliminal to supraliminal) rewards may be processed more fully, involving higher-level cognitive processing. In line with previous research on conscious and unconscious perception (Dehaene et al., 1989), this higher-level cognitive processing is thought to enable more complex cognitive processes and strategic behavioral responses, which could explain why conscious reward processing in some task contexts leads to unique effects.

In experiments, initial (or unconscious) and full (or conscious) reward processing is commonly manipulated by presenting masked reward stimuli (e.g., 1 cent vs. 50 cents coins) either for relatively short (i.e., 17 ms) or relatively long durations (i.e., 300 ms). Subsequent subliminality tests are usually administered to provide evidence that the short presentation of masked reward stimuli renders participants unable to identify the reward value of the stimuli. However, it is questionable whether such tests provide conclusive evidence that short stimulus presentation time prevents conscious perception throughout an experimental task. In fact, there is an ongoing debate about what kinds of subliminality tests are capable of providing sufficient proof for unconscious processing (e.g., see Seth et al., 2008; Sandberg et al., 2010). In the present research, we took a different approach aimed at distinguishing conscious from unconscious reward processing by investigating a situation in which the two types of reward processing are predicted to produce different behavioral effects. Specifically, we test the hypothesis that conscious and unconscious reward value processing differ with regard to taking into account attainability information.

As explained above, when consciously processed rewards vary in attainability, people base their decisions to invest effort on the combination of reward value and attainability. Integration of these two types of information prevents wasting resources on valuable yet unattainable rewards or attainable yet low value rewards (von Neumann and Morgenstern, 1947; Atkinson, 1957, 1964; Vroom, 1964; Brehm and Self, 1989; Camerer and Hogarth, 1999; Bonner and Sprinkle, 2002; Rushworth and Behrens, 2008). Research suggests that the value of a reward and the likelihood that a reward can be attained are initially encoded by distinct subcortical brain networks (Rogers et al., 1999; Dreher et al., 2006; O'Neill and Schultz, 2010), and that the integration of these different signals involves higher cortical processing (Knutson et al., 2005; Tobler et al., 2007; Rushworth and Behrens, 2008; Haber and Knutson, 2009). Therefore, based on the framework outlined above, we predicted that the integration of reward value and attainability requires conscious reward processing. Consequently, when the likelihood of conscious processing is reduced (i.e., by short presentation of rewards), people should fail to integrate reward value and attainability information, resulting in inefficient investment of effort and performance.

The notion that conscious information processing allows for greater integration and more flexible behavioral control is central to several information processing approaches to consciousness (Dehaene and Naccache, 2001; Baars, 2002; Dijksterhuis and Aarts, 2010; Morsella and Bargh, 2010). However, empirical studies have thus far found both support for (e.g., Kunde, 2003; Ansorge et al., 2011) and evidence against (e.g., Lau and Passingham, 2007; Hassin et al., 2009; van Gaal et al., 2010) the hypothesis that conscious information processing plays a unique role in modulating cognitive performance. For instance, studies have shown that subliminally presented stop cues can slow down, but rarely fully inhibit behavioral responses (van Gaal et al., 2008, 2009). Moreover, unconscious stop cues failed to elicit the same globally-distributed and sustained pattern of brain activation observed in response to consciously perceived cues. This work suggests that although unconsciously perceived cues can trigger basic cognitive control processes, conscious perception may enable more efficient and flexible control of behavior (Dehaene and Naccache, 2001).

The present study aims to shed more light on possible advantages of conscious over unconscious reward processing by focusing not only on the initial triggering of cognitive performance by consciously and unconsciously perceived rewards, but on how integration of rewards with attainability information affects performance. Because reward value and attainability information are two distinct aspects of rewards (Brehm and Self, 1989; Liu et al., 2007; Tobler et al., 2007; O'Neill and Schultz, 2010), we expect that full or conscious processing of reward information is necessary to integrate these two types of information and arrive at efficient performance. To test this novel hypothesis, we report behavioral data from two experiments in which we presented participants with high and low-value rewards (coins of 50 or 1 eurocents, respectively) that were instructed to be either attainable or unattainable by successfully performing an active maintenance task. To manipulate the likelihood of conscious vs. unconscious processing, the coins were masked and presented either for relatively long (300 ms) or short (17 ms) durations. We manipulated conscious processing of the reward value rather than attainability information in order to connect our research with previous work on conscious vs. unconscious reward processing (Pessiglione et al., 2007; for an overview see Bijleveld et al., 2012).

Importantly, in order to provide evidence that differences between the effects of long vs. briefly presented rewards are not merely caused by the presentation of attainability information, but are due specifically to differences in the ability to integrate the reward value with attainability information, we manipulated the need for information integration across two experiments. As explained below, Experiment 1 was designed to make integration a requirement for efficient performance, whereas Experiment 2 was designed to eliminate the necessity of integration for efficient performance. Based on the theory that conscious and unconscious reward processing differ in the ability to integrate value and attainability, we expected that conscious and unconscious reward processing would lead to different effects in Experiment 1 but not in Experiment 2. We outline the concrete predictions for the two experiments in more detail below.

Experiment 1 was designed to establish different behavioral effects of quick vs. slowly presented rewards when integrating reward value and attainability was required for efficient responses. This was accomplished by testing performance in response to attainable and unattainable high vs. low value rewards in a full within-subject design. Value and attainability are two distinct sources of performance motivation, and hence performance may be increased by higher reward value, or by the fact that a reward is attainable (e.g., Atkinson, 1957, 1964; Vroom, 1964; Brehm and Self, 1989; Bonner and Sprinkle, 2002). However, when both reward value and attainability vary on a trial-by-trial basis, it is essential to integrate on each trial the two sources of motivation to derive an optimal decision to invest effort (e.g., Anderson, 1971; Brehmer and Joyce, 1988). In this context, we expected that when rewards were presented for a relatively long duration, enabling conscious processing, performance should be enhanced selectively for high value attainable rewards. This result would constitute a conceptual replication of previous work (Atkinson, 1957, 1964; Vroom, 1964; Brehm and Self, 1989; Camerer and Hogarth, 1999; Bonner and Sprinkle, 2002). Examining effects of consciously processed rewards also serves as a control condition to verify that attainability information was clearly and unambiguously processed and that participants were able and motivated to take this information into account.

When rewards are presented for a shorter duration, reducing the likelihood of conscious processing, we predicted a different pattern of results. Without the ability to integrate value and attainability information, participants were expected to invest their effort based either on the high (vs. low) value of a reward, or on the fact that rewards could be gained (vs. not), but not on a combination of both sources of performance motivation. This led to the following predictions: First, the instruction that a reward is attainable vs. unattainable should boost performance. Second, the perception of high vs. low value rewards should likewise boost performance. Most importantly, without the ability to integrate value with attainability information, perception of high value rewards should boost performance, even when it is clear that the reward is unattainable. In summary, we expected that performance would be boosted independently by the fact that a reward can be earned and the presentation of a high value coin. This should result in main effects of reward value and attainability.

Experiment 2 was designed to provide attainability information without requiring trial-by-trial integration with reward value. To do so, we manipulated the attainability of rewards between participants. The idea behind this was that when attainability information constitutes a stable dimension for an individual (cf. Waltz et al., 1999), participants can employ a general response strategy that is valid on every trial without requiring integration of incoming information. More specifically, when rewards are always attainable, participants can respond efficiently based on reward value alone. Likewise, when rewards are always unattainable, the decision to invest effort can be based on this information alone, neglecting the reward value. Hence, in Experiment 2 participants were expected to perform better for high vs. low attainable rewards regardless of whether rewards were presented for long or short duration (e.g., Pessiglione et al., 2007; Zedelius et al., 2011b). Moreover, we expected participants to perform equally well for unattainable rewards, regardless of whether rewards were presented for long or short duration.

## Experiment 1

### Method

#### Participants and design

Participants were 41 undergraduate students (28 female). A 2 (presentation duration: long vs. short) × 2 (value: low vs. high) × 2 (attainability: attainable vs. unattainable) within-participants design was employed.

#### Procedure

**Figure 1:**
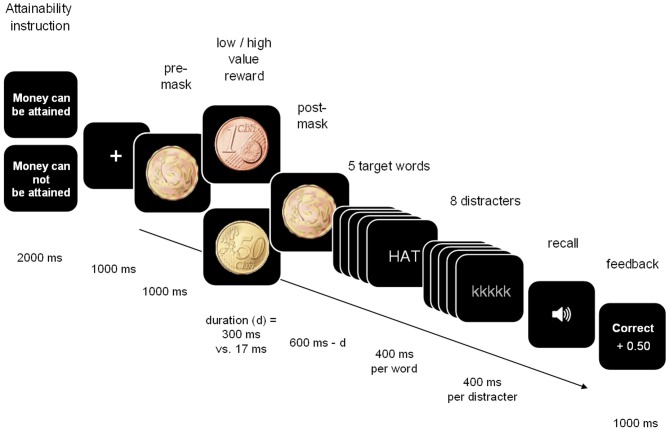
**Overview of the procedure of Experiment 1**.

Participants performed a verbal active maintenance task in which they were asked to actively maintain word spans of five one-syllable nouns in WM while inhibiting mild distraction during a short delay interval (see Conway et al., 2005; Zedelius et al., 2011b). For an overview of the procedure including pictures of the reward- and masking stimuli, see Figure 1. Participants were told that on every trial of the maintenance task, coins were presented that served as rewards for correct responses. Participants were further told that the coins would sometimes be “difficult to perceive” (referring to the short presentation condition). Furthermore, participants learned that the money would not always be attainable, and that they would be paid the amount of rewards earned on attainable reward trials at the end of the experiment.

Each trial started with the message, “Reward can be attained” or “Reward cannot be attained”, presented for 2000 ms. Following a previously developed procedure, a fixation cross was then shown on the screen for 1000 ms, followed by a mask (a scrambled picture of both 1 and 50 cents coins) for 1000 ms, followed by the presentation of a 1 cent or 50 cents coin. The coin was presented for either 300 ms (long presentation condition) or 17 ms (short presentation condition) and followed by a post-mask presented for 600 ms, minus the duration of the coin. Subliminality of the stimuli was tested in a separate detection task with 25 different participants. On each trial, participants saw a coin (1 cent vs. 50 cent), presented in the same way as in the experiment (17 ms in between masks). After each coin, participants indicated the value of the coin. A *t*-test indicated identification of the coins was no better than chance (*M* = 0.51, *SD* = 0.11), *t* (24) = 0.43, *n.s*. (see Bijleveld et al., 2009 for another subliminality check of this procedure)^1^.

After the coin presentation, the target words were presented for 400 ms per word, with an inter-word interval of 200 ms. The presentation of the target words was followed by a delay period during which mildly distracting letter strings were shown for 800 ms each intermitted by intervals of 500 ms. After this delay period, participants were asked to verbally report the target words. Performance was considered correct when all five words were correctly reported. The order in which the words were reported could be arbitrary (see Zedelius et al., 2011a, for the validity of this measure). Finally, accuracy feedback and, for attainable reward trials, the amount obtained was shown. The task consisted of 56 randomly presented trials (seven repetitions per condition). After the experiment, participants were paid the amount of money they had earned throughout the task. The experiment was conducted according to institutional guidelines and approved by the local ethics committee.

### Results and discussion

**Figure 2:**
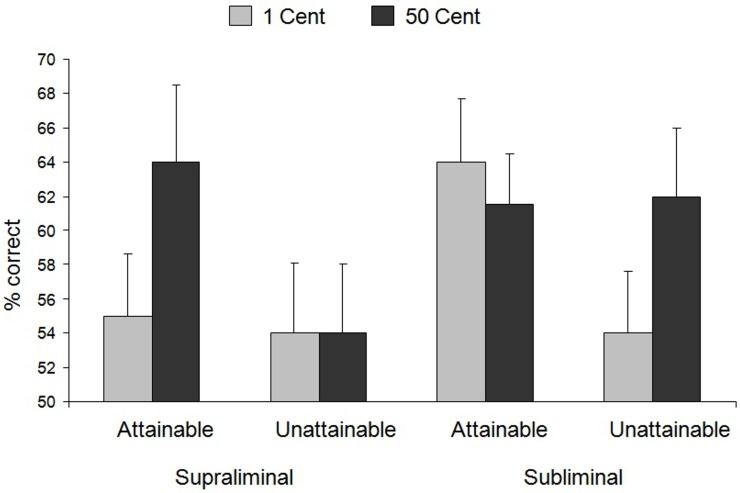
**Results of Experiment 1**. Mean and standard error of the percentage of correct trials as a function of reward value, presentation duration, and attainability. Error bars = SE.

To test our hypothesis that the duration of reward presentation affects the integration of reward value and attainability information, the proportion of correct trials^2^ was subjected to repeated-measures ANOVA according to the experimental design. The analysis revealed a main effect of attainability, [*F*_(1, 40)_ = 4.63, *p* = 0.04, η^2^_p_ = 0.10], qualified by the predicted three-way interaction of presentation duration × value × attainability, [*F*_(1, 40)_ = 6.20, *p* = 0.02, η^2^_p_ = 0.13] (see Figure 2). To test the hypothesis that in the long presentation condition effort is selectively increased when rewards are both high and attainable, we performed a specific contrast comparing performance on the long presented high value attainable reward trials with performance on the other trials within the long presentation condition. This contrast was significant, *F*_(1, 40)_ = 8.07, *p* = 0.007, η^2^_p_ = 0.17, indicating that performance was indeed selectively increased for high value attainable rewards. This result is in line with classic theories of motivation that predict enhanced effort and performance only when rewards are both valuable and attainable (e.g., Hull, 1943; Brehm and Self, 1989).

In the short presentation condition we expected that reward value and attainability information would boost performance independently, resulting in main effects of reward value and attainability. However, contrary to this prediction, we found no main effect of reward value, *F*_(1, 40)_ = 1.25, *n.s.*, and no main effect of attainability, *F*_(1, 40)_ = 2.52, *n.s*. Instead, we found a marginally significant interaction of reward value and attainability, [*F*_(1, 40)_ = 3.50, *p* = 0.07, η^2^_p_ = 0.06]. Further inspection of this interaction with simple effects analyses indicated that performance increased in response to high vs. low value rewards when these were unattainable, [*F*_(1, 40)_ = 4.52, *p* = 0.04, η^2^_p_ = 0.10]. Thus, consistent with the prediction outlined in the introduction, high reward value of briefly presented coins boosted performance even when the reward was unattainable (note that the clarity of the attainability information can be inferred from the conscious reward condition). Moreover, and consistent with the prediction that the opportunity to obtain a reward would boost performance in the short presentation condition, we found that performance was increased in response to attainable compared to unattainable low value presented rewards, [*F*_(1, 40)_ = 6.31, *p* = 0.02, η^2^_p_ = 0.14]. However, and contrary to our expectation, we found that performance on attainable reward trials was equally high for both high and low value coins, *F* < 1. The absence of a boosting effect of high value in this latter comparison likely explains why we did not obtain the expected two main effects.

How can we explain the unexpected finding that performance in the briefly presented attainable reward condition was unaffected by the value of the rewards? First, we can rule out that the value of the rewards was not encoded in the short presentation condition. This is attested by the effect of reward value in the briefly presented *unattainable* reward condition. We can also rule out that the absence of an effect of value in the attainable reward condition was merely due to a lack of statistical power. That is, previous research testing the effects of attainable rewards on performance using the same experimental task and procedure (Zedelius et al., 2011b; low distraction condition) indicates that the effect of briefly presented rewards is of small to medium size (d_z_ = 0.41; Cohen, 1988). According to a power analysis using the statistical software G^*^Power 3.1 (Faul et al., 2009), the chance of detecting an effect of this size with a desired statistical power of minimally 0.80 at an alpha level of 0.05 requires a sample of at least 39 participants, which we exceeded in the present experiment.

Accordingly, we think that there may be a theoretical explanation for why performance in the briefly presented attainable reward condition was unaffected by reward value. As argued above, without the ability to integrate reward value and attainability information, the mere fact that a reward is attainable should cause participants to recruit effort to perform well. This hypothesis was confirmed by the fact that performance was boosted in response to low value attainable rewards within the short presentation condition. The question is whether performance can be increased even further by the presentation of an attainable high value reward. The absence of a main effect of value suggests that this may not be possible. One straightforward explanation for the absence of a value effect in this condition is that the mere opportunity to gain a reward already promoted maximal investment of effort, leaving no room for an additive effect of high reward value on performance. This explanation is consistent with other research showing that factors that independently increase motivation for action (e.g., testosterone and reward cues) do not produce additive effects, probably because motivation is already boosted to its limits by one factor alone, minimizing the contribution of a second source of motivation (see Aarts and van Honk, 2009). This argument implies that we should obtain an effect of value when variation in attainability is not a source of performance enhancement. This issue is addressed in Experiment 2.

Because this experiment is the first examination of conscious and unconscious reward effects under varying attainability conditions within the same task, one question that comes to mind is whether performance in response to attainable and unattainable rewards was influenced by reward attainability on the previous trial. Although we did not predict this, it is an interesting possibility that should be taken into account in light of evidence for performance adjustments instigated by specific trial sequences (e.g., Kunde, 2003; Boy et al., 2010; Ansorge et al., 2011). Therefore, we explored whether attainability sequence (i.e., whether attainability on trial n was the same vs. different from trial *n*–1) affected the results reported above. Specifically, we performed an additional repeated-measures ANOVA with the factors reward value, presentation duration, attainability, and attainability sequence. The results showed no main effect of attainability sequence, and no interaction effects of attainability sequence with any of the above reported factors (all *F*s < 1.14). These findings indicate that the differential effects of attainable and unattainable rewards were not affected by the presence or absence of the chance to attain a reward on the previous trial.

Another question that may be raised is whether different effects of long vs. short presentation of attainable and unattainable rewards may be driven by feedback learning. Although participants received accuracy feedback on all trails, feedback about the amount of reward obtained could only be given on attainable reward trials. Could differences in feedback between the attainable and unattainable conditions account for the effects reported above? We do not expect this for a number of reasons: first, we used reward stimuli that were familiar to participants from everyday life so that the reward value likely did not require learning. Second, on attainable reward trials, the coins presented at the beginning of a trial were 100% indicative of the amount of reward to be earned given optimal performance. Thus, and unlike in some other studies (e.g., Knutson et al., 2005; Dreher et al., 2006; Bjork and Hommer, 2007; Tobler et al., 2007), there was no ambiguity about the amount that could be earned on each trial. Moreover, an explanation in terms of added learning on attainable reward trials would be inconsistent with the finding that the briefly presented high vs. low rewards selectively increased performance in the unattainable reward condition, where no feedback was given about the reward value. However, to statistically rule out that learning played a role in driving the above effects, we performed an additional analysis including factors of the experimental design and the additional factor block (i.e., first vs. second half of the trials). The results showed that block did not interact significantly with any of the reported effects, and, most importantly, it did not qualify the above mentioned three way interaction of reward value, presentation duration, and attainability, [*F*_(1, 40)_ = 1.57, *ns*]. Consequently, differences in feedback learning from attainable and unattainable reward trials do not seem to account for different effects of long vs. briefly presented attainable and unattainable rewards.

If our predictions outlined in the introduction are correct and conscious compared to unconscious reward processing enables greater integration of value and attainability information, differences between the effects of conscious and unconscious rewards should vanish when people do not need to integrate this information. To test this hypothesis in Experiment 2, we varied attainability information between, rather than within, participants such that the rewards were either always attainable or always unattainable. When rewards are always attainable, only the value dimension is important to boost performance, and no information integration is required. Therefore, we predicted performance to be enhanced by both long and short presentation of high compared to low value rewards. In contrast, when rewards are always unattainable, and thus never worth the effort, incoming information about the reward value becomes irrelevant. In this case, neither kind of reward should affect performance.

## Experiment 2

### Method

#### Participants and design

Participants were 33 undergraduates (24 female). The design was a 2 (presentation duration: long vs. short) × 2 (value: low vs. high) × 2 (attainability: attainable vs. unattainable) mixed design with duration and value as within-participants factors and attainability as between-participants factor.

#### Procedure

The same WM task was used as in Experiment 1, with the only difference that reward attainability instructions varied between participants. In the attainable reward condition, participants were told that the coins displayed throughout the task were rewards that could be attained for accurate performance. In the unattainable reward condition, participants were told that the coins had functioned as rewards for performance in a previous experiment, but that in this Experiment the rewards were unattainable. In this condition, participants received a flat rate of 5 euros for their participation in the experiment. The experiment was conducted in accordance with institutional guidelines and approved by the local ethics committee.

### Results and discussion

**Figure 3:**
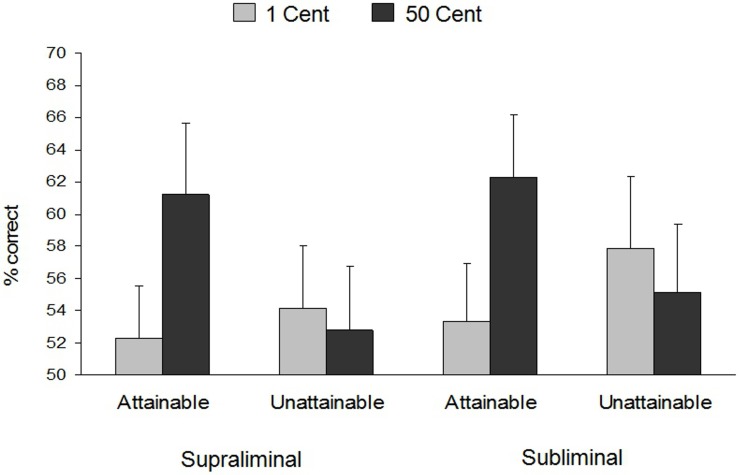
**Results of Experiment 2**. Mean and standard error of the percentage of correct trials as a function of reward value, presentation duration, and compensation. Error bars = SE.

The proportion of correct trials was subjected to an ANOVA according to the design. There were no main effects of presentation duration, *F*_(1, 31)_ = 1.15, *p* = 0.29, reward value, *F*_(1, 31)_ = 2.16, *p* = 0.15, or attainability, *F* < 1. However, we did find the predicted interaction between value and attainability, [*F*_(1, 31)_ = 5.62, *p* = 0.02, η^2^_p_ = 0.15]. This interaction was not qualified by a three-way interaction with presentation duration, *F* < 1. Simple effects analyses showed, first that when rewards were attainable, both long and short presentation of high compared to low value rewards increased performance, [*F*_(1, 31)_ = 6.75, *p* = 0.01, η^2^_p_ = 0.18] (see Figure 3). This finding is a direct replication of previous studies (e.g., Pessiglione et al., 2007; Bijleveld et al., 2010; Capa et al., 2011; Zedelius et al., 2011b). This replication is particularly important in light of the unexpected finding from Experiment 1 that performance for briefly presented attainable rewards was unaffected by the reward value. As argued above, in a context of varying opportunity to attain rewards (Experiment 1), the instruction that a reward was attainable caused participants to invest maximal effort in response to briefly presented low value rewards, leaving no room for further improved by high reward value. The present findings from the second experiment show that the performance boost for briefly presented low value attainable rewards does not occur when attainability is a fixed factor within participants.

The results further showed that, when rewards were unattainable, performance for both long and briefly presented rewards was unaffected by the reward value, *F* < 1. This finding confirms our prediction that short presentation of unattainable high value rewards does not lead to enhanced performance when integration of reward value and attainability information is unnecessary for efficient responding. When it is clear that rewards are never attainable, and hence high value rewards are never worth investing extra effort, people can employ the same general and predefined response strategy throughout the task. That is, they can prepare to ignore the value of rewards even before the rewards are presented. Such a strategy might alter their perception of the rewards such that high value rewards are no longer perceived as valuable or rewarding (Delgado et al., 2008; Staudinger et al., 2009). As such, results of Experiment 2 converge well with work showing that when rewards are irrelevant for behavioral responses, initial reward processing in the subcortical reward system is unaffected by the reward value (Bjork and Hommer, 2007).

In summary, the results from Experiment 2 suggest that both conscious and unconscious reward processing can boost performance efficiently when there is no requirement to integrate value and attainability information. In light of Experiment 1, Experiment 2 provides further evidence that conscious compared to unconscious reward processing promotes the integration of incoming reward value and attainability information.

## General discussion

The aim of the present study was to test whether conscious compared to unconscious processing of rewards leads to more efficient cognitive task performance based on the successful integration of reward value and attainability information. To examine this question, we first examined the situation in which the need for integration was relatively high by varying value and attainability information on a trial-by-trial basis (Experiment 1). In line with traditional theories of motivation and decision making (e.g., Hull, 1943; von Neumann and Morgenstern, 1947; Atkinson, 1957, 1964; Vroom, 1964; Brehm and Self, 1989), we found that when coins were presented for a relatively long duration, and could thus be consciously perceived, performance increased selectively for valuable and attainable rewards. In contrast, brief presentation of the coins led to rather inefficient effort investment and performance. First, and most stunning, participants worked harder for high compared to low rewards despite their conscious knowledge that the rewards were unattainable. Second, when participants were instructed that rewards were attainable, performance was increased regardless of the reward value. These findings suggest that brief presentation of rewards, which reduces the likelihood of conscious processing, causes failure to integrate reward value and attainability information. Moreover, our data suggest that in the absence of integration, high reward value and information that a reward is attainable do not improve performance in an additive way. Instead, people invest maximal effort in response to either source of motivation.

The fact that performance was more efficient when reward information was presented for a relatively long duration speaks to the hypothesis that conscious awareness enables processes that lead to more strategic behavior. This finding converges well with the framework outlined in the introduction, according to which initial or unconscious reward processing can directly facilitate task performance, but full or conscious reward processing is needed to modulate performance strategically (Bijleveld et al., 2012). Support for the direct facilitation of performance through rewards comes from neuroscience research showing that the value of rewards is first encoded in a subcortical reward network, including most prominently the ventral striatum (VS) (Phillips et al., 2007; Salamone et al., 2009). The VS is also responsible for translating the reward value into effort by projecting to frontal cortical areas, such as the dorsolateral prefrontal cortex, which modulate executive control processes (Aston-Jones and Cohen, 2005; Liljeholm and O'Doherty, 2012; Schmidt et al., 2012). This may explain why unconsciously perceived rewards can facilitate effortful cognitive performance. However, according to the framework (Bijleveld et al., 2012), conscious awareness of rewards allows for more complex, higher-level cognitive processing (see also Dehaene et al., 1989). Such higher level processing likely includes activation of the medial and orbital prefrontal cortex, regions that are involved in evaluating the likelihood that a reward can be attained (Rogers et al., 1999; Knutson et al., 2005; O'Neill and Schultz, 2010). This may explain why consciously processed rewards lead to more efficient effort investment based on the combination of reward value and attainability information.

Further evidence for the crucial role of consciousness in integrating value and attainability information stems from Experiment 2, where we show that long and short presentation of rewards lead to parallel effects on performance when integration of value and attainability was irrelevant. That is, irrespective of presentation duration of the reward information, participants performed better for relatively high attainable rewards, but performance was similar for high and low rewards when these were unattainable. An interesting question raised by this latter finding is whether the coins were still perceived as rewarding when they are always unattainable. Although money is generally desirable (Lea and Webley, 2006), it is possible that the perception of money as a performance reward depends on the potential of attaining it (cf. Biner and Hannon, 1988; Richter and Gendolla, 2006). Further research is therefore needed to determine whether cognitive task performance is boosted by unconscious reward cues as a function of the actual or perceived rewarding property of the cues.

It is important to note that a few recent studies have shown that conscious and unconscious rewards can sometimes have different effects on cognitive control task performance. For instance, it has been shown that conscious, but not unconscious high rewards impair performance when they are presented during the execution of an active maintenance task, probably due to distraction (Zedelius et al., 2011b). Furthermore, while unconsciously presented monetary rewards were shown to reduce the attentional blink effect (assessed by the rapid serial visual presentation task; Raymond et al., 1992), conscious rewards augmented the attentional blink effect resulting from the (normatively learned) tendency to concentrate too much on task stimuli when one knows that rewards are relatively high (Bijleveld et al., 2011). These previous studies point to an advantage of unconscious reward processing in boosting cognitive control performance. The present study contributes to this research by demonstrating that the advantageous or disadvantageous effects of conscious vs. unconscious rewards depend on the ability to combine relevant information to arrive at efficient cognitive task performance.

The results of the present study have important implications for current debates about the role of consciousness in motivation and decision making (Dijksterhuis and Aarts, 2010; Baumeister et al., 2011). That is, even though information integration is sometimes proposed to be dependent on conscious processing (e.g., Dehaene and Naccache, 2001; Baars, 2002; Dijksterhuis and Aarts, 2010; Morsella and Bargh, 2010; however, see Mudrik et al., 2011), conscious and unconscious processing are rarely compared directly to test differences in integration. Employing a paradigm where conscious and unconscious reward processing can directly be compared, the present study suggests that conscious awareness plays a crucial role in the integration of reward value and attainability information to arrive at an optimal decision about whether it is worthwhile to invest effort. This ability to integrate different types of reward related information may not be constrained to value and attainability information. Even when valuable rewards are attainable, people may judge them not worth the effort, for instance because they are very hard to get or because they are attainable only after a considerable delay (e.g., Kivetz, 2003; Raynolds, 2006). Such judgments imply the combination of reward value with information about effort and time requirements (Ballard and Knutson, 2009). Although the exact mechanisms behind these judgments go beyond the current research, our findings suggest that they may benefit from conscious awareness of rewards.

## Directions for future research

The present findings raise interesting questions for future research. First, given that (attainable and unattainable) unconsciously perceived rewards can motivate people to work, this leads to the question of how people might experience this motivation. Although the framework outlined above makes a qualitative distinction between conscious and unconscious reward processing, this framework does not imply that rewards perceived outside of conscious awareness can never gain access to consciousness, or affect conscious experience in any way. For instance, when people become motivated by unconscious rewards, they may become aware of this motivation, either indirectly, by observing their own behavior, or more directly, by noticing potential changes in their mood or arousal which may be related to their motivated behavior (e.g., Carver and Scheier, 1990; Knutson et al., 2005; Chartrand et al., 2010). Although this topic goes beyond the scope of the current investigation, it remains an interesting direction for future research. Within the present research, however, there is no evidence that potential downstream effects of unconscious rewards on conscious experience could help the strategic control of efficient effortful performance.

Another interesting topic for future research is how conscious expectations with regard to the value of unconsciously processed rewards affect performance and motivation. For instance, would a person work harder for an unconsciously perceived low value attainable reward when he or she consciously expects it to be of high, rather than low value? In the light of the present studies, we can only speculate about this issue. In the present study, when attainability varied throughout the task, participants based their decisions to invest effort either on high value of a reward, or the fact that the reward was attainable. This suggests that people are most strongly influenced by information that triggers motivational behavior. Information that should reduce motivated behavior (i.e., the fact that a high value reward was not attainable, or that an attainable reward was of low value) appeared to have less impact. Therefore, we would predict that conscious expectancies related to reward value could overrule the effects of unconsciously perceived rewards when people expect a high value reward, but unconsciously perceived high value should drive behavior when people expect to work for a low value reward. It would be interesting to test these predictions in future work, for instance by manipulating the (perceived) ratio of high to low value rewards.

## Conclusion

The present study extended recent research on conscious and unconscious reward pursuit by addressing the issue of how people deal with unattainable rewards. The findings from two experiments with different experimental designs suggest that conscious perception of rewards enables people to integrate the value of monetary rewards with fluctuating attainability information. Thus, while consciousness of rewards is certainly not necessary to boost cognitive task performance, it appears to be crucial to arrive at efficient effort investment when confronted with attainable and unattainable rewards.

### Conflict of interest statement

The authors declare that the research was conducted in the absence of any commercial or financial relationships that could be construed as a potential conflict of interest.

## Footnotes

^1^The fact that coin identification was at chance level calls for an objective assessment that the coins were indeed presented. For this purpose, we ran the experiment again on one of the computers used for data collection, and recorded 18 trials of the 17 ms coin presentation using a camera with a slow motion feature (a Sony NX4), enabling recording of the coin presentation at the rate of 240 frames per second. Inspection of the recordings showed that the coin was visible in each recording.

^2^Because earning rewards (on attainable reward trials) was contingent on recalling all words correctly, accuracy was operationalized as the correct recall of all five words. Because this demand was clear from the instructions, we reasoned that the total number of words recalled per trial would be a suboptimal performance measure in the present experiment. (For a more detailed discussion of this argument, see Zedelius et al., 2011a). However, for both studies, the pattern of results for the total number of words recalled resembled that of the accuracy data. Specifically, in Experiment 1, analysis of the total number of correctly recalled words yielded a marginally significant main effect of attainability, *F*_(1, 40)_ = 3.56, *p* = 0.07, η^2^_p_ = 0.08, which was qualified by a marginally significant three-way interaction of attainability × reward value × exposure, *F*_(1, 40)_ = 3.00, *p* = 0.09, η^2^_p_ = 0.07. When rewards were attainable, performance was higher for high (*M* = 4.32, *SD* = 0.59) compared to low value rewards (*M* = 4.08, *SD* = 0.72) in the long presentation condition, *F*_(1, 40)_ = 5.18, *p* = 0.03, η^2^_p_ = 0.11, but performance was equally high for high (*M* = 4.33, *SD* = 0.52) and low (*M* = 4.31, *SD* = 0.46) value rewards in the short presentation condition, *F*_(1, 40)_ = 0.12, *n*.s. When rewards were unattainable, performance did not differ for high (*M* = 4.16, *SD* = 0.69) and low value rewards (*M* = 4.16, *SD* = 0.61) in the long presentation condition, *F*_(1, 40)_ < 0.001, *n*.s. Performance appeared to be somewhat higher for high (*M* = 4.19, *SD* = 0.72) compared to low (*M* = 4.12, *SD* = 0.60) value rewards in the short presentation condition, although this latter increase was not statistically significant (*F*_(1, 40)_ = 0, 71, *n*.s.). In Exp. 2, we found a significant interaction of attainability x reward value, *F*_(1, 31)_ = 5.18, *p* = 0.03, η^2^_p_ = 0.14, indicating performance was not influenced by the reward value in the unattainable reward condition (high reward value: *M* = 4.16, *SD* = 0.50; low reward value: *M* = 4.20, *SD* = 0.44), *F*_(1, 31)_ = 0.38, *n*.s., but performance was increased for high (*M* = 4.32, *SD* = 0.40) vs. low value rewards (*M* = 4.13, *SD* = 0.58) in the attainable reward condition, *F*_(1, 31)_ = 6.33, *p* = 0.02, η^2^_p_ = 0.17, and this effect did not differ for the long and short presentation conditions, *F*_(1, 31)_ = 0.21, *n.s*.
